# RING RASH IN AN INFANT: UNMASKING NEONATAL LUPUS

**DOI:** 10.51894/001c.122894

**Published:** 2024-08-30

**Authors:** Aashir Siddique, Kripa Thakur, Landon Barlow, Michelle Gallagher

**Affiliations:** 1 College of Osteopathic Medicine Michigan State University

## 36

### INTRODUCTION

Neonatal lupus (NLE) is a rare congenital condition secondary to autoantibodies passing from the mother to the fetus. These autoantibodies, particularly anti-Ro (SSA) and anti-La (SSB), are commonly found in systemic lupus erythematosus (SLE). Maternal-fetal transmission of these antibodies can lead to the many clinical features of NLE, including a non-pruritic rash localized to the face, trunk, and extremities, cytopenia, hepatomegaly, and first-to-third-degree AV block. The extracutaneous findings of NLE make it a high-mortality condition with potential lifelong complications.

### CASE DESCRIPTION

We present a three-month-old male who developed large erythematous plaques soon after birth that were localized to the face, back, and torso. The patient was born with no complications and received proper prenatal checkups. The appearance of the rash led to a presumptive diagnosis of seborrheic dermatitis and erythema multiforme. The infant was treated with Selenium shampoo, but it provided no improvement after 10 days. Further history taking revealed SLE in the mother, which was being treated with hydroxychloroquine. On physical examination, the patient was in no acute distress, and hepatomegaly was absent. Cutaneous examination revealed multiple large ring-like reddish/purple plaques with associated scaling and erythema. The combination of clinical history, physical examination, and blood work led to the diagnosis of NLE in the patient. Namely, the patient’s autoantibody analysis showed significantly elevated positive titers for anti-SSA/Ro (>240.9 U/mL), and anti-SSB/La (154.0 U/mL). The patient’s CBC and hepatic function tests were within normal limits. Cardiology was consulted which ruled out heart block in the infant. Importantly, other dermatological mimickers of NLE, which can also present with ringlike rashes, were differentiated on physical exam. Upon diagnosis, the patient was treated with 2.5% hydrocortisone topical ointment. Follow-up examination eight weeks later showed marked improvement in the patient’s lesions.

### DISCUSSION/CONCLUSIONS

This case illustrates how NLE can often be misdiagnosed as other, more benign dermatological conditions. Clinicians need to be able to recognize the etiology, signs, and symptoms of NLE. With early recognition of the characteristic rash of NLE, and a subsequent prompt diagnosis, neonates can be effectively treated preventing severe cardiac complications which can ultimately be fatal in misdiagnosed patients.

